# The impact of technology on promoting physical activities and mental health: a gender-based study

**DOI:** 10.1186/s40359-023-01348-3

**Published:** 2023-09-29

**Authors:** Yangyang Liu, Hongxue Zhang, Ruilin Xu

**Affiliations:** 1 School of Physical Education (Main Campus), Zhengzhou University, Zhengzhou, 450001 China; 2 School of Physical Education, Zhengzhou University, Zhengzhou, 450044 Henan Province China

**Keywords:** Technology, Barriers, Gender-based differences, Mental health, Physical activities, Motivations

## Abstract

**Background:**

Physical inactivity is a significant public health concern globally, associated with an increased risk of chronic diseases and detrimental effects on both physical and mental health. Technologically based interventions have emerged as a potential solution to promote physical activity engagement and improve mental health outcomes. However, understanding the effectiveness of these interventions and the role of gender in their outcomes is essential for developing tailored strategies.

**Objective:**

This study aims to examine the effectiveness of technologically based interventions in promoting physical activity and improving mental health outcomes, with a specific focus on gender differences.

**Methodology:**

This study employed a three-phase mixed methods research design. Phase one was an experimental phase where 300 participants were randomly assigned to intervention or control groups. The intervention group received a technologically based physical activity intervention, while the control group did not. Physical activity levels and mental health outcomes were assessed before and after the intervention. Phase two involved qualitative interviews with a subset of participants (n = 20) from the intervention group. These interviews explored motivations and barriers to physical activity, aiming to uncover personal factors influencing engagement. Thematic analysis was used to identify recurring themes. Phase three utilized a quantitative survey to compare motivations and barriers between males and females. The survey, administered to a larger sample, included participants from both intervention and control groups. It assessed various factors and allowed for a quantitative comparison of gender differences.

**Findings:**

findings indicated that the intervention improved the mental health and physical activities level of the intervention groups. Findings also there are 8 motivations for and barriers to using technology in physical activities. Male and females’ scores on some of the motivations and barriers were statistically significant.

**Conclusion:**

technology plays an important role in improving the mental health and physical activities of adults. Findings can be used by health care centers, digital psychologists, and physical trainers.

**Supplementary Information:**

The online version contains supplementary material available at 10.1186/s40359-023-01348-3.

## Introduction

Participating in consistent physical activity yields a wide array of advantages, such as the mitigation of cardiovascular ailments [1], more effective weight control [2], diminished chronic pain [3], heightened bone density and physical aptitude [4, 5], augmented muscle strength, and decreased risk of mortality [6, 7]. In addition, engaging in physical activity has been associated with various positive psychological outcomes, including enhanced mental well-being and overall quality of life [8]. It has also been found to contribute to the reduction of stress and anxiety levels [9, 10], lower the likelihood of experiencing depression [11], and promote better sleep quality [12]. Additionally, there exists a positive correlation between engagement in physical activity and academic achievement among individuals enrolled in higher education institutions [13]. Physical activity and exercise have a strong correlation with psychological well-being and social welfare [14]. Hence, it is imperative for individuals to engage in a consistent regimen of physical activities. The integration of technology into individuals’ daily routines and physical endeavors is feasible. The prevalence of technology, ranging from smartphones and laptops to gaming consoles and smartwatches, has had a profound impact on various aspects of our lives, including our daily routines, professional endeavors, and recreational activities. Nevertheless, the growing utilization of technology has resulted in a predominantly inactive way of life, posing a significant issue for public health [15]. Regular physical activity is imperative for the maintenance of a healthy lifestyle and the prevention of chronic ailments such as obesity, type 2 diabetes, and cardiovascular diseases [16]. Conversely, the influence of physical activity and sedentary behavior on happiness and mental health is a significant consideration in our lives [17, 18]. Hence, it is imperative to examine the correlation between technology-mediated physical activities, subjective well-being, and psychological well-being.

The rationale for conducting a gender-based study on the role of technology in promoting physical activities and mental health stems from the growing recognition of both gender-specific disparities in health outcomes and the ubiquitous integration of technology in modern lifestyles [1–5]. As society becomes increasingly reliant on digital devices and platforms, it is crucial to explore how these technologies impact the physical activity levels and mental well-being of individuals, with attention to potential gender-based variations. This study seeks to provide insights into whether and how technology can serve as a tool to mitigate gender-specific health disparities by investigating the ways in which men and women engage with and benefit from technological interventions aimed at enhancing physical activity and mental health [6, 9]. Understanding these dynamics can inform targeted interventions and policy measures to harness technology’s potential for promoting healthier lifestyles and reducing gender-based health inequities. Moreover, including a gender-based analysis is essential to uncover potential disparities in the impact of technology on physical activity and mental health, ensuring that interventions can be tailored to address unique needs and preferences of both men and women. This approach recognizes the significance of gender as a relevant factor in promoting equitable health outcomes in the context of technology-driven interventions, ultimately fostering more inclusive and effective strategies for all.

The aim of this study is to investigate the influence of technology on the promotion of physical activity and its subsequent effects on the mental health and overall well-being of employees, with a particular focus on employing a gender-based analytical approach. The qualitative aim of the study was exploring the participants’ perceptions of the motivations for using technology-based physical activity interventions in the workplace and the barriers they face when using technology-based physical activities. This investigation specifically concentrates on exploring potential disparities related to gender. Identifying and addressing these disparities is critical for bridging the existing gap in knowledge and understanding. The results of this study will offer significant contributions to workplace health promotion initiatives and policies that seek to increase physical activity and improve mental health and well-being. Special attention will be given to addressing the unique requirements and perspectives of individuals of various genders, further emphasizing the need to close the gender-based gap in this vital research area.

### Research questions

In line with the research objectives, the following research questions were stated:

1. Is there any statistically significant difference between the physical activity levels and mental health of male and female employees who use technology-based physical activity interventions in the workplace?
2. What motivates male and female employees to use technology-based physical activity interventions in the workplace?
3. What barriers do male and female employees face when using technology-based physical activity interventions in the workplace?
4. Is there any statistically significant difference between males’ and females’ perceptions of motivations and barriers to using technology-based physical activity interventions in the workplace?

## Review of literature

### Physical activities and mental health outcomes

A multitude of rigorously conducted studies have consistently demonstrated the favorable influence of physical activity on diverse facets of mental health. Various perspectives and methodologies in research provide evidence to support the proposition that individuals who participate in consistent physical activity are less prone to developing clinical depression. For example, in a study conducted by Camacho et al. [19], it was observed that individuals who maintained a sedentary lifestyle or engaged in minimal physical activity exhibited increased likelihood of developing depression over a period of 9 years. Paffenbarger and Leung [20] conducted a study which revealed that individuals who participated in sustained and moderate levels of physical activity experienced a notable decrease in the likelihood of developing depression when compared to those who engaged in low levels of physical activity.

The extant body of literature, encompassing both qualitative narratives and quantitative meta-analytical reviews, offers additional corroboration for the aforementioned findings. Numerous studies have consistently shown the beneficial impact of physical exercise on mental well-being across diverse populations, including adolescents [21] and individuals spanning various age groups [22, 23]. Numerous studies have been conducted to investigate the influence of physical exercise on the alleviation of anxiety symptoms. Among these studies, aerobic activities such as running have demonstrated a moderate level of efficacy. Moreover, the inclusion of physical exercise in intervention programs has been linked to a modest decrease in anxiety levels. Nevertheless, research examining the effects of physical activity on the psychological and physiological responses to stress has produced inconsistent findings, possibly as a result of difficulties in measuring these variables.

Boutcher [24] and Etnier et al. [25] have conducted studies investigating the impact of exercise on cognitive functions, specifically reaction time, memory, and fluid intelligence. Boutcher’s cross-sectional studies have revealed a positive association between physical fitness and cognitive performance among older adults. However, experimental studies have yielded inconclusive findings, as certain intervention studies have reported improvements in cognitive function, while others have not observed statistically significant changes. The correlation between physical activity and self-esteem has been examined by Fox [26] as well as Spence and Poon [27]. The results suggest that there is a limited and inconclusive relationship between physical activity and overall self-esteem, as demonstrated by Spence’s meta-analysis which reported a small average effect size. According to the findings of Fox, a significant proportion of randomized controlled trials and controlled trials conducted since 1971 have reported positive improvements in self-esteem, with approximately 50% of these studies demonstrating such outcomes.

In their study, Kandola et al. [28] conducted a comparative analysis to examine the impact of aerobic training and mental training on cognitive function. The results indicated that both forms of training, whether practiced individually or in combination, yielded positive enhancements in cognitive function. In a study conducted by Telenius et al. [29], the effects of a high-intensity exercise program on balance, muscle strength, and reduced apathy in nursing home residents with dementia were examined, revealing positive outcomes.

### Gender differences in physical activities

Rates of obesity, diabetes, and cardiovascular disease (CVD) have continued to rise across populations in many Western countries and other parts of the world [30–33] Previous literature reports a higher rate of obesity in females [34], leading subsequent research to focus on gender differences in physical activity [35]. Moreover, findings from other research suggest that females are less physically active than males [36–42]. For example, Brand et al. (36) found that even among adolescents engaged in ‘high’ levels of moderate-to-vigorous physical activity, males were more active than females. The reported deficit in physical activity in females has been attributed to a range of social and cultural factors, including the complex relationships between physical activity, feminine ideals, and body-image factors [35].

Further research by Martins, Marques, Sarmento, and da Costa [43] has highlighted that most studies on perceptions of physical activity have focused on adolescent females. Their systematic review identified various barriers to physical activity, including attitudes toward physical activity, motivation, perceptions of competence and body image, fun, influence of friends, family, and physical education teachers, and environmental physical activity opportunities [43]. Fun was frequently cited as a reason for female physical activity engagement [43, 44], and it is important to consider participants’ perceptions of fun. Research has shown that fun is related to specific physical activities, such as yoga [45]. Additionally, it is important for activities to be challenging yet non-competitive, with autonomy and social support from family members and a high perception of competence being important factors [45, 46].

Although recent research has identified the use of electronic devices as a barrier to physical activity participation [47], the role of mobile technology in mediating gender differences in physical activity remains uncertain. Gender differences in the use of mobile devices, such as smartphones and tablets, have been observed, with technology often designed to cater to the needs of male gamers [48, 49]. Studies have found significantly higher gender differences in gaming time among adolescents, indicating greater involvement of boys compared to girls [50]. Furthermore, mobile devices can offer incentives that influence physical activity levels, with access to fitness apps promoting an active lifestyle [51, 52]. However, excessive dependence on mobile technology for gaming and social networking can contribute to a sedentary lifestyle [53]. Therefore, device use may serve as both a barrier and a facilitator of physical activity.

### Research method

The present study utilized a mixed-methods approach in order to address the research inquiries. Mixed-methods research refers to a research design that encompasses the collection and analysis of both quantitative and qualitative data within a singular study [54]. This methodology facilitates a more holistic comprehension of the research inquiry by integrating the advantages of both quantitative and qualitative methodologies. The initial stage of the investigation involved the implementation of a quantitative pretest/posttest design, wherein participants were randomly assigned to either control or experimental groups. The objective of this phase was to assess the efficacy of technology-driven interventions promoting physical activity within the workplace. The participants were assigned in a random manner to either a control group or an experimental group. The experimental group was administered technology-based physical activity interventions, whereas the control group did not receive such interventions. The physical activity levels of the participants were assessed prior to and following the intervention through the utilization of a validated questionnaire. The collected data underwent analysis utilizing both descriptive and inferential statistics in order to assess the efficacy of the interventions in promoting higher levels of physical activity.

The subsequent stage of the investigation involved the utilization of a qualitative research approach to examine the participants’ perspectives on physical activities facilitated by technology. The objective of this phase was to enhance comprehension of the participants’ encounters with the technology-based interventions for physical activity. The participants were subjected to interviews employing open-ended questions, and the data gathered were subsequently subjected to thematic analysis. The themes that were identified in the study offer valuable insights into the participants’ experiences with the interventions, encompassing their perceived advantages as well as the obstacles they encountered.

The third phase of the study encompassed a survey that aimed to investigate the disparities in perceptions between males and females regarding the incentives and obstacles associated with utilizing technology-based interventions for physical activity. The objective of this phase was to ascertain whether there existed any gender disparities in the motivations and obstacles experienced by the participants in utilizing the interventions. The survey was distributed to participants of both genders, and the collected data was subjected to analysis employing descriptive and inferential statistical methods in order to ascertain any notable disparities between the two groups.

The utilization of a mixed-methods approach in this study facilitated a more comprehensive comprehension of the research inquiries. The quantitative phase of the study yielded empirical data regarding the efficacy of physical activity interventions that utilized technology, while the qualitative phase offered a comprehensive exploration of participants’ personal encounters with these interventions. The survey conducted during the third phase facilitated the examination of gender disparities in the motivations behind and obstacles to utilizing the interventions. By integrating both quantitative and qualitative methodologies, this research endeavor successfully presented a comprehensive depiction of the efficacy of technology-driven physical activity interventions within professional settings.

### Participants

During the initial stage of the investigation, the sample comprised of 300 individuals, encompassing both male and female employees hailing from diverse colleges and departments of Zhengzhou University. The participants were selected through convenience sampling. In the initial stage of the study, a quantitative pretest/posttest design was employed, utilizing randomized control and experimental groups. The participants were randomly allocated to either the control or experimental group. In each group, there were 150 participants (75 males and 75 females). The experimental group was provided with physical activity interventions that were based on technology, whereas the control group did not receive any such interventions. The distribution of participants in this phase was equal between males and females. In the subsequent phase of the study, a qualitative research approach was employed to investigate the participants’ perspectives on physical activities facilitated by technology. A total of 20 participants were selected using purposive sampling. The rationale for the sample selected for qualitative phase was data saturation which occurred when the 20th participant was interviewed. The individuals involved in this stage were equally divided between males and females, and they had all been exposed to technology-driven interventions for physical activity within the workplace. The sample size for the current phase was comparatively smaller than that of the preceding and subsequent phases, as the primary objective was to acquire more comprehensive and detailed insights into the participants’ experiences. In the third phase all selected participants were enlisted for this phase. The sample consisted of an equal distribution of male and female participants, all of whom had received technology-based physical activity interventions in their workplace. They were asked to attempt the questionnaire related to motivations and barriers of technological -based intervention.

### Instruments

Distinct instruments were employed for various stages of the investigation. During the initial stage of the study, data from the participants was collected using two distinct measures. The initial assessment utilized the Short Form Questionnaire of International Physical Activities (SE-IPAQ), a scale consisting of seven items designed to evaluate self-reported physical activities. This questionnaire assesses various aspects such as the quantity of moderate- and vigorous-intensity physical activity, walking behavior, and sedentary time engaged in by participants within the preceding seven-day period. The validity and internal consistency of this measure have been established in prior research.

In the initial stage, the mental health scale was employed as the second measure. The measurement of participants’ mental health was conducted using the General Mental Health Questionnaire-28 (GHQ-28), which was developed by Goldberg and Hillier in 1979. The questionnaire utilized in this study was piloted and validated by Shayan et al. [55]. The GHQ-28 is comprised of four dimensions that assess the somatic symptoms, anxiety and insomnia, social dysfunction, and depression experienced by the participants. There are a total of seven items within each dimension, which are assessed using a Likert scale.

Furthermore, the initial phase of the study incorporated the utilization of two sports applications as a means to deliver technology-driven interventions for physical activity. The initial application, known as MyFitnessPal, primarily centers its functionality on the monitoring and recording of nutritional intake. Additionally, it offers a comprehensive compilation of exercises and workout routines that users can input and monitor to assess their progress over a period of time. The subsequent application examined is the 7 min Workout, a mobile application that offers expedient and effective exercise routines that can be tailored to suit the individual’s fitness proficiency and personal inclinations.

During the subsequent stage of the research, a structured interview checklist was employed to gather qualitative data pertaining to the participants’ perspectives on physical activities that utilize technology. The interview checklist comprised a series of open-ended inquiries that were formulated to investigate the participants’ encounters with workplace interventions involving physical activity facilitated by technology (Appendix A). The questions were intentionally crafted with flexibility and adaptability in mind, aiming to accommodate the participants’ responses and facilitate a comprehensive investigation into their experiences. The utilization of an interview checklist during this phase facilitated a heightened level of individualized and comprehensive comprehension regarding the viewpoints of the participants.

During the third phase of the study, data was collected on the disparities between male and female perspectives regarding motivations for and obstacles to utilizing technology-based physical activity interventions. This was achieved through the implementation of a researcher-developed questionnaire comprising 13 items (Appendix A). The development of the questionnaire was informed by the findings obtained from the investigation of research questions 2 and 3. The instruments were specifically developed to assess different dimensions pertaining to the participants’ motivations for and obstacles to utilizing the interventions. These dimensions include perceived advantages, perceived difficulties, and the inclination to sustain the use of the interventions. The researcher conducted a validation process on the questionnaire in order to establish its validity and reliability. The findings from the factor analysis indicated that the loading factors for all items surpassed the threshold of 0.75, indicating a strong relationship between the items and their respective factors. Additionally, the internal consistency of the factors was found to be above 0.83, suggesting a high level of reliability within the factors.

### Procedure

The research was carried out in three distinct stages in order to examine the efficacy of technology-driven physical activity interventions within a corporate environment. During the initial stage, individuals were enlisted and granted utilization of two technology-driven interventions for physical activity, namely MyFitnessPal and 7 min Workout. Participants were instructed to utilize the interventions for a duration of four weeks. At the conclusion of this four-week period, data pertaining to their levels of physical activity and mental well-being were gathered. The Short Form Questionnaire of International Physical Activities (SE-IPAQ) and the General Mental Health Questionnaire-28 (GHQ-28) were employed for this purpose.

During the subsequent stage, individuals who had successfully completed the intervention period of four weeks were extended an invitation to partake in a semi-structured interview. Participants were presented with open-ended inquiries regarding their encounters with technology-based physical activity interventions. The interviews were then recorded in audio format and transcribed verbatim to facilitate subsequent analysis.

During the third phase of the study, individuals who had successfully finished the four-week intervention period were requested to participate in a survey. This survey, created by the researchers, comprised 13 items that aimed to assess the participants’ perceived motivations and barriers towards utilizing technology-based physical activity interventions. Additionally, the survey aimed to explore potential gender differences in these perceptions. The researcher conducted a validation process on the questionnaire prior to its distribution among the participants. Subsequently, the collected data were analyzed to obtain a deeper understanding of the variables that could potentially impact the efficacy of technology-driven physical activity interventions.

### Data analysis

#### Phase 1

The data collected from the SE-IPAQ and GHQ-28 were analyzed using ANOVA to examine the effects of the physical activity interventions on participants’ physical activity levels and mental health, respectively. It is worth noting that the assumptions for ANOVA test such as homogeneity of variances (Levene’s test) and normality assumption were checked. Specifically, repeated measures ANOVA was used to compare participants’ physical activity and mental health scores before and after the four-week intervention period. Post-hoc tests, such as Bonferroni or Tukey, were conducted to identify significant differences between the pre-and post-intervention scores. In addition, descriptive statistics, such as means and standard deviations, were used to provide an overview of participants’ physical activity levels and mental health at each time point and to identify any trends or patterns in the data.

### Phase 2

The audio recordings of the interviews were transcribed verbatim and analyzed using thematic analysis. Two researchers independently reviewed the transcripts and coded the data into themes based on the participants’ responses to the interview questions. The researchers then compared and discussed their findings to reach a consensus on the final themes and sub-themes.

### Phase 3

The data collected from the researcher-developed questionnaire were analyzed using descriptive statistics to examine participants’ responses to each item. Independent sample t-tests were used to compare responses between male and female participants. Exploratory factor analysis was conducted to identify underlying factors related to participants’ motivations for and barriers to using technology-based physical activity interventions.

### Findings

#### Phase 1

The mean scores of the male and female participants were submitted to an ANOVA test. Descriptive statistics are presented in Table 1.

**Table 1:** Descriptive statistics for physical activity levels and mental health scores

		Male - Control	Male - Intervention	Female - Control	Female – Intervention
Variable	Time	M (SD)	M (SD)	M (SD)	M (SD)
Physical activity levels	Pre-intervention	3.25 (0.89)	3.39(1.1)	3.3(0.98)	3.4(0.98)
	Post-intervention	3.4(0.83)	5.4(1.1)	3.53(0.86)	5.3(1.1)
Mental health	Post-intervention	3.5 (0.96.)	3.6(1.1)	3.63(0.83)	3.50(0.83)
	Pre-intervention	3.6 (0.82)	4.3 (1.2)	3.70(0.86)	4.5(1.1)

Note: Values are presented as means (standard deviations). F-values and p-values are from repeated measures ANOVA.

For physical activity levels, the results indicate that both male and female participants in the intervention group showed a greater increase in physical activity levels from pre- to post-intervention compared to the control group. Specifically, male participants in the intervention group had a mean increase of 1.9 (SD = 1.1) in SE-IPAQ score, while male participants in the control group had a mean increase of 0.15 (SD = 0.89). Similarly, female participants in the intervention group had a mean increase of 1.8 (SD = 1.1) in SE-IPAQ score, while female participants in the control group had a mean increase of 0.23 (SD = 0.98). Results also show that there was a significant difference in mental health scores for female participants between the control and intervention groups. Specifically, female participants in the intervention group had a mean decrease of 1.0 (SD = 0.83) in GHQ-28 score from pre- to post-intervention, while female participants in the control group had a mean decrease of 0.2 (SD = 0.86). Results of the ANOVA test are presented in Table 2.

**Table 2:** Repeated measures ANOVA results for physical activity levels and mental health scores

Variable	Source	SS	df	MS	F	p
Physical activity levels	Time	794,676.25	1	794,676.25	30.8	< 0.001
	Gender	36,645.23	1	36,645.23	1.4	0.244
	Time × Gender	7,016.53	1	7,016.53	0.3	0.584
Mental health	Time	45.31	1	45.31	9.2	0.003
	Gender	57.23	1	57.23	2.3	0.136
	Time × Gender	29.16	1	29.16	5.9	0.019

Note: SS = sum of squares, df = degrees of freedom, MS = mean square, F = F-value.

As seen in Table 2, there is a notable primary effect of time (F (1, 49) = 30.8, p < 0.001) in relation to physical activity levels. This suggests that the intervention had a substantial impact on enhancing physical activity levels. The results indicate that gender did not have a statistically significant main effect on physical activity levels (F (1, 49) = 1.4, p = 0.244). Additionally, there was no significant interaction effect between time and gender (F (1, 49) = 0.3, p = 0.584). These findings suggest that the intervention had a comparable impact on physical activity levels for both male and female participants. The analysis of mental health scores revealed a statistically significant main effect of time (F (1, 49) = 9.2, p = 0.003), suggesting that the intervention had a notable impact on enhancing mental health. The analysis revealed that gender did not have a statistically significant main effect on the outcome variable (F (1, 49) = 2.3, p = 0.136). However, there was a statistically significant interaction effect between time and gender (F (1, 49) = 5.9, p = 0.019), suggesting that the impact of the intervention on mental health varied for male and female participants.

### Findings of phase 2

The second phase of the study explored the motivations for employees to use technology-based physical activity interventions and barriers to using technology-based physical activity interventions.

### Motivations

The interviews with the participants were thematically coded and eight types of motivations were extracted, each theme is exemplified as follows.

#### Health and fitness goals

One of the motivations for using technology-based physical activity interventions at work reported by participants in the study was to achieve their health and fitness goals. They expressed that using these interventions helped them keep track of their workouts, monitor their progress, and stay motivated to reach their fitness goals. Participant C’s statements further exemplify this theme, as they stated, “I use the fitness app on my phone because it helps me keep track of my workouts and stay motivated to reach my fitness goals” and “I like using the fitness tracker because it helps me monitor my progress and make adjustments to my workout routine.“ These quotes highlight how technology-based physical activity interventions can motivate individuals to achieve their health and fitness goals by providing them with tools to track their progress and stay motivated.

#### Convenience and accessibility

Another motivation for using technology-based physical activity interventions at work reported by participants was the convenience and accessibility they provided. Participants expressed that these interventions were a convenient and accessible way to engage in physical activity at work. Participant D’s statements further illustrate this theme, as they stated, “I don’t have time to go to the gym after work, so using the fitness app during my lunch break is a convenient way for me to get some exercise” and “The fitness tracker is always with me, so I can use it whenever I have a few spare minutes at work to get some physical activity.“ These statements highlight how the convenience and accessibility of technology-based physical activity interventions can motivate individuals to engage in physical activity at work, as they provide an easy and accessible way to incorporate physical activity into their daily routines.

#### Social support

Participants reported that using technology-based physical activity interventions with colleagues or friends at work provided social support and motivation to be physically active. They expressed that engaging in physical activity interventions with others at work created a sense of community and encouraged them to be more active. Participant E’s statements further exemplify this theme, as they stated, “We have a step challenge at work, and it’s fun to see how I’m doing compared to my colleagues” and “I like using the fitness app with my friend at work because we can encourage each other and hold each other accountable.“ This finding clarifies how social support from colleagues or friends can motivate individuals to use technology-based physical activity interventions in the workplace. By engaging in physical activity interventions with others, individuals can create a sense of camaraderie and accountability, which can motivate them to be more physically active.

#### Incentives and rewards

The participants in the study indicated that the provision of incentives or rewards for engaging in technology-based physical activity interventions in the workplace served as a motivating factor for increasing their levels of physical activity. The participants conveyed that the provision of incentives and rewards yielded a palpable advantage in their involvement with technology-based physical activity interventions. Participant F expressed that their company implements a wellness program that provides incentives for utilizing fitness trackers, which in turn serves as a source of motivation for them to increase their usage. Additionally, Participant F conveyed their appreciation for the opportunity to accumulate points through the utilization of the fitness application, which can subsequently be redeemed for rewards such as gift cards or fitness-related merchandise. Hence, through the provision of incentives for the utilization of these interventions, corporations can effectively motivate individuals to participate in physical activity, thereby enhancing their holistic well-being and health.

#### Tracking progress

The participants in the study indicated that the utilization of technology-based interventions for physical activity enabled them to monitor their progress and observe advancements over a period of time. This aspect emerged as a prevalent motivation for their adoption of such interventions. The individuals conveyed that these interventions afforded them the opportunity to track their physical activity and assess their progress towards their objectives. Participant N’s statement provides additional evidence that supports the prevailing theme. They express their preference for utilizing the fitness application due to its ability to display the number of steps taken and calories burned, enabling them to track their progress over a period of time. This quotation substantiates the notion that interventions centered around technology can effectively stimulate individuals to participate in physical activity by equipping them with a means to monitor their advancement and observe enhancements over a period of time. Consequently, through monitoring their advancements, individuals are able to gauge the extent of their achievements and experience a sense of motivation to persist in their participation in physical exercise.

#### Personalization

The participants in the study indicated that technology-driven interventions for physical activity, which could be tailored to their specific preferences and requirements, were found to be more motivating for their engagement. This finding aligns with the general trend observed among participants, where motivation was consistently identified as a key factor influencing their utilization of such interventions. The participants conveyed that the option to personalize these interventions according to their individual workout regimen and objectives engendered a heightened sense of involvement and motivation in utilizing them. The statement made by Participant B serves as an additional illustration of the aforementioned theme, as they expressed their preference for the ability to personalize the fitness application according to their individual exercise regimen and objectives, thereby enhancing their motivation to utilize it. This quotation suggests that the incorporation of personalization into physical activity interventions through technology can serve as a motivating factor for individuals. By granting individuals a sense of autonomy and control over their fitness journey, personalization can encourage their active participation. By tailoring these interventions to suit their personal preferences and specific needs, individuals can experience a heightened sense of engagement and motivation, thereby increasing the likelihood of sustained usage.

#### Competition

The participants expressed that the utilization of technology-based interventions for physical activity within a competitive framework, such as engaging with colleagues or friends, proved to be both motivating and enjoyable. This aspect emerged as a recurring motivation for their adoption of such interventions. The participants conveyed that their engagement and motivation to engage in physical activity were heightened through their involvement in step challenges or other competitive activities facilitated by technology-based interventions. The statement made by Participant C serves as an additional illustration of the prevailing theme, as they expressed their enjoyment in engaging in step challenges alongside their colleagues due to the enjoyable and motivating nature of observing who can accumulate the highest number of steps. The present theme underscores the role of competition in stimulating individuals to participate in physical activity interventions that incorporate technology, thereby offering them an enjoyable and captivating means to enhance their level of physical activity. Engaging in competitive activities has been found to enhance individuals’ motivation to partake in physical exercise, thereby facilitating improvements in their overall health and fitness levels.

#### Stress relief

The participants in the study indicated that the utilization of technology-based physical activity interventions in the workplace served as a means of alleviating stress and enhancing their sense of energy and concentration. This emerged as a prevalent motivation for engaging in such interventions. The participants conveyed that their involvement in physical activity interventions utilizing technology afforded them a means to alleviate stress and experience heightened levels of energy throughout the remainder of their workday. Participant D’s statement provides additional evidence to support the prevailing theme, as they expressed their preference for utilizing the fitness application during their midday break. They noted that this practice aids in their relaxation and enhances their energy levels, thereby positively impacting their productivity for the remainder of the workday. The aforementioned discovery underscores the efficacy of employing technology-driven interventions for physical activity, as they effectively incentivize individuals to partake in exercise by affording them a means to alleviate stress and augment their energy levels. Participating in physical activity can enhance an individual’s emotional state and cognitive abilities, thereby yielding advantageous outcomes for their general health and overall state of well-being.

### Barriers employees face when using technology-based physical activity interventions in the workplace

Interviews were content analyzed and the following themes were extracted:

#### Lack of time

Upon thematic coding of the data, the first identified barrier to using technology-based physical activity interventions at work was a lack of time. Participants reported that due to their busy work schedules and numerous deadlines and meetings, they often found it challenging to take a break for physical activity. Quotations from Participant 10 exemplify this theme, as he stated, “I’m usually too busy with work to take a break for physical activity, and I have a lot of deadlines and meetings, so I don’t have much time during the workday to use the fitness app.“ This highlights how time constraints can hinder the adoption of technology-based physical activity interventions in the workplace.

#### Lack of motivation

The second identified barrier to using technology-based physical activity interventions at work was a lack of motivation, as reported by the participants. They expressed that they often lacked the drive to engage in physical activity using technology-based interventions while at work. Quotations from Participant 7 further illustrate this theme, as they stated, “I just don’t feel motivated to use the fitness app at work” and “I’m usually too tired or stressed to think about physical activity during the workday.“ These quotes highlight how a lack of motivation can act as a deterrent to adopting technology-based physical activity interventions in the workplace, as participants may not feel inclined to use these interventions due to a lack of interest or energy.

#### Lack of support

Another identified barrier to using technology-based physical activity interventions at work was a lack of support from colleagues or supervisors, as reported by the participants. Participants expressed that they did not feel supported when trying to use these interventions due to a lack of encouragement or interest from their colleagues or supervisors. Quotations from Participant 7 further illustrate this theme, as they stated, “My supervisor doesn’t seem to think physical activity is important, so I don’t feel supported when I try to use the fitness app at work” and “None of my colleagues use the fitness app, so I don’t feel like I have any support or motivation to use it either.“ These quotes highlight how the lack of support and motivation from colleagues or supervisors can impact an individual’s willingness to adopt technology-based physical activity interventions in the workplace. Without peer support and encouragement, individuals may feel demotivated and less likely to engage in these interventions.

#### Workplace culture

The workplace culture, specifically a sedentary work environment, was identified as another barrier to using technology-based physical activity interventions at work, as reported by the participants. They expressed that the physical setup of the workplace made it challenging to think about physical activity during working hours. Participant A’s statements further illustrate this theme, as they stated, “Our office is set up so that we’re sitting all day, which makes it hard to think about physical activity” and “There’s no culture of physical activity at work, so it feels weird to use the fitness app during work hours.“ These quotes depict how the workplace culture, which promotes a sedentary lifestyle, can act as a barrier to adopting technology-based physical activity interventions. Without a culture of physical activity in the workplace, individuals may feel uncomfortable or out of place engaging in physical activity interventions, and the physical environment may not support their efforts to do so.

#### Technical issues

Technical issues, including difficulties using or accessing technology-based physical activity interventions, were also identified as a barrier to their use, as reported by the participants. Participants expressed those technical challenges hindered their ability to engage in physical activity interventions using technology. Participant D’s statements further exemplify this theme, as they stated, “The fitness tracker is really complicated to use, so I don’t use it very often” and “The fitness app doesn’t work on my phone, so I can’t use it even if I want to.“ These quotes highlight how technical issues can pose challenges for individuals trying to use technology-based physical activity interventions. Difficulties with the technology itself, such as complicated user interfaces or compatibility issues, may prevent individuals from engaging in these interventions.

## Findings of phase 3

The males’ and females’ mean scores on motivations and barriers were statistically compared; results are presented in Table 3.

**Table 3:** T-tests for comparing male and female participants’ perceptions of motivations and barriers

Variables	Males Mean	bmc SD	Females Mean	Females SD	T	df	P
Health and fitness goals	3.75	1.1	4.00	0.9	-0.99	298	0.336
Convenience and accessibility	3.66	0.9	3.8	0.88	-0.49	298	0.632
Social support	4.2	1.1	3.5	0.98	-2.19	298	0.041*
Incentives and rewards	3.6	0.9	3.8	N/A	-0.66	298	0.518
Tracking progress	3.8	0.9	3.9	0.69	-0.43	298	0.674
Personalization	4.2	1.1	3.8	0.95	1.15	298	0.264
Competition	4.2	0.9	3.7	0.89	1.85	298	0.080
Stress relief	3.6	0.98	3.8	0.79	-0.61	298	0.548
Lack of time	3.2	0.65	3.7	0.63	-2.24	298	0.039*
Lack of motivation	2.5	0.5	2.6	0.63	-0.52	298	0.609
Lack of support	2.5	0.63	3.1	0.89	-2.21	298	0.041*
Workplace culture	2.7	0.63	3.6	0.97	-3.13	298	0.005**
Technical issues	2.6	0.96	2.68	0.86	-0.29	298	0.776

Note: * indicates statistical significance at p < 0.05

According to the data presented in Table 3, the variable “Health and fitness goals” exhibits distinct differences between males and females. Specifically, the mean value for males is 3.75, (SD = 1.1). In contrast, females have a higher mean value of 4.00(SD = 0.9). The findings indicate that female participants exhibited marginally higher levels of motivation towards health and fitness objectives within the workplace compared to their male counterparts. Additionally, the responses provided by females displayed less variability in comparison to the responses of males.

In relation to the variable “Convenience and accessibility,“ the average score for males is 3.66, (SD = 0.9). Conversely, the average score for females is 3.8(SD = 0.88). The findings suggest that women expressed marginally greater levels of motivation towards technology-based physical activity interventions in the workplace, specifically in terms of convenience and accessibility. However, it is important to note that the observed difference is relatively minor. In relation to the variable “Social support,“ the average value for males is 3.5, (SD = 1.1). Conversely, mean score of females is 4.2(SD = 0.98). These findings suggest that women expressed notably greater levels of motivation for seeking social support in technology-based physical activity interventions within the workplace compared to men. Additionally, it was observed that women’s responses exhibited less variability in comparison to men’s responses.

The variable “Incentives and rewards” exhibits a mean of 3.6 (SD = 0.9) for males. However, the mean for females cannot be determined as it is unavailable due to missing data. Hence, it is not possible to derive any meaningful interpretation regarding the association between this variable and gender. In relation to the variable “Tracking progress,“ it is observed that males have a mean score of 3.8, (SD = 0.9. On the other hand, females have a mean score of 3.9 (SD = 0.69). These findings indicate that females tend to report slightly higher levels of motivation in tracking the progress of technology-based physical activity interventions in the workplace compared to males. However, it is important to note that the difference between the two groups is relatively small.

In relation to the variable “Personalization,“ the average score for males is 4.2, (SD = 1.1). Conversely, the average score for females is 3.8, (SD = 0.95. The findings suggest that males exhibited significantly greater levels of motivation towards personalizing technology-based physical activity interventions in the workplace compared to females, although there was considerable variability in responses among both genders.

In relation to the variable “Competition,“ the average value for males is 4.2, (SD = 0.9). Conversely, the average value for females is 3.7, (SD = 0.89). The findings suggest that male participants exhibited significantly greater levels of motivation for engaging in technology-based physical activity interventions in the workplace compared to their female counterparts. However, it is worth noting that there was a considerable degree of variability in the responses from both genders. In regards to the variable “Stress relief,“ it is observed that the average value for males is 3.6 (SD = 0.98). On the other hand, the average value for females is 3.8, (SD = 0.79). These findings indicate that women exhibited marginally greater levels of motivation for utilizing technology-based physical activity interventions as a means of stress relief in the workplace, in comparison to men. However, it is important to note that the observed difference is relatively minor. The t-tests were performed under the assumption of unequal variances, specifically utilizing Welch’s t-test, with a predetermined significance level of 0.05. The findings of the study suggest that there were no statistically significant disparities observed between males and females across the majority of the variables examined. However, a notable exception was observed in relation to social support, where females reported significantly higher levels compared to males (p < 0.05).

The descriptive statistics reveal that in relation to the variable “Lack of time,“ the male participants exhibited a mean score of 3.2, (SD = 0.65). Conversely, the female participants displayed a mean score of 3.7, (SD = 0.63). The results of the t-test indicate that there is a statistically significant difference in mean scores between males and females (t(18) = -2.24, p = 0.039*). This suggests that females tend to report a higher frequency of experiencing a lack of time for physical activity in the workplace compared to males. In relation to the variable “Lack of motivation,“ it was observed that males exhibited a mean score of 2.5 (SD = 0.5). Conversely, females displayed a mean score of 2.6, (SD = 0.63). The results of the t-test indicate that there is no statistically significant difference in the mean scores (t(18) = -0.52, p = 0.609) between males and females in terms of their motivation levels for engaging in physical activity in the workplace.

In relation to the variable “Lack of support,“ it was observed that males exhibited a mean score of 2.5, (SD = 0.63. Conversely, females displayed a mean score of 3.1, along (SD = 0.89). The results of the t-test indicate that there is a statistically significant difference in mean scores (t(18) = -2.21, p = 0.041*), suggesting that females tend to report a higher frequency of experiencing a lack of support for physical activity in the workplace compared to males. In relation to the variable “Workplace culture,“ it was observed that males exhibited a mean score of 2.7, (SD = 0.63). Conversely, females displayed a mean score of 3.6, (SD = 0.97. The results of the t-test indicate that there is a statistically significant difference in mean scores (t(18) = -3.13, p = 0.005**), suggesting that females perceive a more supportive workplace culture for physical activity compared to males. In relation to the variable “Technical issues,“ it was observed that males exhibited a mean score of 2.6, (SD = 0.96). Conversely, females displayed a mean score of 2.68, along (SD = 0.86). The results of the t-test indicate that there is no statistically significant difference in mean scores between males and females in relation to technical issues associated with physical activity in the workplace (t(18) = -0.29, p = 0.776). This suggests that the experiences of males and females in this regard are not significantly different.

## Discussion

With regard to the first research question, findings confirmed the effectiveness of the technology based physical intervention in promoting the employees’ mental health and physical partitivities. In the same direction, numerous studies have examined the effectiveness of technology-based interventions in promoting physical activity and improving mental health outcomes. A systematic review and meta-analysis conducted by Fanning, et al., [56] found that technology-based interventions were effective in increasing physical activity levels and improving mental health outcomes in both healthy and clinical populations. Similarly, a meta-analysis by Stephenson, et al., [57] found that mobile phone, internet, and wearable device interventions were effective in promoting physical activity and reducing sedentary behavior.

The current study’s findings are consistent with these previous studies, as the technology-based physical activity intervention had a significant positive effect on physical activity levels and mental health in both male and female participants. However, the current study also found a significant interaction effect of time × gender on mental health, suggesting that the intervention may have differential effects on mental health for male and female participants. One potential explanation for this gender difference in the effects of the intervention on mental health may be related to differences in the types of activities that male and female participants engage in. For example, research has suggested that male participants may be more likely to engage in high-intensity physical activities, such as weightlifting or running, which have been shown to have a positive effect on mental health [58]. In contrast, female participants may be more likely to engage in lower-intensity physical activities, such as yoga or Pilates, which have also been shown to have a positive effect on mental health [59]. The current study did not collect data on the specific types of physical activities that participants engaged in, so further research would be needed to explore this hypothesis.

In conclusion, the current study’s findings are consistent with previous research that has shown the effectiveness of technology-based interventions in promoting physical activity and improving mental health outcomes. However, the study also highlights the potential for gender differences in the effects of these interventions on mental health, and further research is needed to explore these differences and identify potential underlying mechanisms.

The use of technology-based physical activity interventions in the workplace has become increasingly popular in recent years, with many individuals using apps and fitness trackers to monitor and increase their physical activity levels. Understanding the motivations and barriers to using these interventions is important in promoting their use and effectiveness in improving health outcomes. Motivations for using technology-based physical activity interventions in the workplace include health and fitness goals, convenience and accessibility, social support, incentives and rewards, tracking progress, personalization, and competition. Participants in a study by Althoff et al. [60] reported that the use of technology-based physical activity interventions helped them achieve their health and fitness goals, monitor their progress, and stay motivated to reach their fitness goals. They also expressed that the convenience and accessibility of these interventions provided an easy and accessible way to incorporate physical activity into their daily routine. The social support provided by colleagues or friends was also found to be a significant motivator, creating a sense of camaraderie and accountability [61].

Incentives and rewards were also found to be motivating factors, as they provided participants with a tangible benefit for engaging in physical activity interventions using technology [60]. Tracking progress and personalization were also found to be important motivators, as they provided individuals with control over their fitness journey and the ability to monitor their progress over time [61]. Finally, competition was found to be a motivator, providing individuals with a fun and engaging way to be physically active [59].

The second and the third research questions addressed the motivations for and barriers the employees faced using technological-based devices for doing physical activities. We found that despite the many motivators for using technology-based physical activity interventions in the workplace, there are also several barriers to their use. These barriers include lack of time, cost, privacy concerns, and technological issues. Participants in a study by Althoff et al. [60] reported that lack of time was a significant barrier, as they struggled to find time to engage in physical activity interventions during the workday. Cost was also a barrier, with some individuals expressing concerns about the cost of purchasing fitness trackers or gym memberships. Privacy concerns were also cited as a barrier, as some individuals were uncomfortable with the idea of sharing their physical activity data with their employer or colleagues. In addition to these barriers, technological issues were also found to be a significant barrier to the use of technology-based physical activity interventions in the workplace. Participants in a study by Finkelstein et al. [30] reported that technological issues, such as difficulty using the apps or fitness trackers, were a significant barrier to their use.

Therefore, it can be postulated that technology-based physical activity interventions have the potential to be effective tools for promoting physical activity and improving health outcomes in the workplace. Motivators for their use include health and fitness goals, convenience and accessibility, social support, incentives and rewards, tracking progress, personalization, and competition. However, to promote their effectiveness, barriers such as lack of time, cost, privacy concerns, and technological issues must be addressed. Employers and researchers must take these factors into account when designing and implementing technology-based physical activity interventions in the workplace.

The results of this study provide valuable insights into the motivations of males and females for technology-based physical activity interventions in the workplace. The findings suggest that some gender differences exist in motivation for certain aspects of these interventions, but not in others. Specifically, females reported slightly higher levels of motivation for health and fitness goals and convenience and accessibility, but the differences were relatively small. However, females reported significantly higher levels of motivation for social support than males, indicating that they perceive this factor as more important for their engagement in technology-based physical activity interventions in the workplace. These findings are consistent with previous research on gender differences in motivation for physical activity. For example, a study by van Stralen et al. [62] found that social support was a more important predictor of physical activity for women than for men. Another study by van den Berg, et al. [63] found that women were more likely to use technology-based interventions for physical activity than men and that social support was a significant predictor of their engagement in these interventions. These findings suggest that social support may be a particularly important factor to consider when designing workplace interventions aimed at increasing physical activity among female employees.

The finding that males reported significantly higher levels of motivation for personalization and competition than females is somewhat surprising, given that previous research has suggested that these factors may be more important for males than females [e.g., 64]. However, the responses for both genders were relatively variable, indicating that personalization and competition may not be universally important factors for all individuals. This highlights the importance of tailoring workplace interventions to the specific needs and preferences of individual employees. In general, the findings of this study have important implications for workplace interventions aimed at increasing physical activity among employees. The results suggest that social support is a particularly important factor to consider when designing interventions for female employees, while personalization and competition may be more important for males. By taking these gender differences into account, employers can design interventions that are more effective and engaging for all employees, leading to improved health and well-being in the workplace.

Results also showed some interesting gender differences in experiences related to physical activity in the workplace. The results show that there are significant differences between males and females in their experiences of time and support for physical activity in the workplace, as well as workplace culture. However, there are no significant differences between males and females in their experiences of motivation or technical issues. The finding that females report experiencing a lack of time for physical activity in the workplace more often than males is consistent with previous research. For example, a study by Hershman et al. [65] found that women who work full-time report less leisure-time physical activity than men, and that lack of time is a significant barrier to physical activity for women in particular. The present study’s finding that females report experiencing a lack of support for physical activity in the workplace more often than males is also consistent with previous research. A study by Gao et al. [66] found that social support, including support from coworkers and supervisors, is a significant predictor of physical activity in the workplace.

The finding that females report a more supportive workplace culture for physical activity than males is also consistent with previous research. A study by Xu et al. [67] found that workplace culture, including social norms and support for physical activity, is associated with physical activity levels among employees. However, the finding that there are no significant differences between males and females in their experiences of motivation or technical issues related to physical activity in the workplace is somewhat surprising, given that previous research has suggested that these factors may be important barriers to physical activity for both genders [40]. Overall, these findings have important implications for workplace interventions aimed at promoting physical activity and improving the health and well-being of employees. The results suggest that interventions should be tailored to address the specific barriers and challenges faced by each gender, including lack of time and support for females, and workplace culture for both genders. By addressing these barriers and promoting a supportive workplace environment for physical activity, employers can help to improve the health and well-being of their employees and reduce healthcare costs associated with physical inactivity.

This study identifies several key motivators for using technology-based interventions, including health and fitness goals, convenience, social support, and progress tracking. These factors align with established drivers of physical activity engagement. Barriers to using these interventions are also recognized, such as lack of time, cost, privacy concerns, and technological issues. These challenges reflect common obstacles encountered when implementing such programs in a workplace setting. The gender-based analysis reveals that females tend to prioritize social support more than males, indicating its significance in their engagement with these interventions. Surprisingly, males express a higher motivation for personalization and competition, suggesting the need for tailored approaches based on individual preferences. More importantly, the study underscores the importance of considering gender differences when designing workplace interventions to enhance physical activity. Recognizing these distinctions can lead to more effective and inclusive programs, ultimately improving employee health and well-being.

## Conclusions and implications

The present investigation revealed that a physical activity intervention utilizing technology yielded positive results in terms of enhancing physical activity levels and enhancing mental health outcomes among participants of both genders. The intervention group exhibited a notable rise in physical activity levels from pre- to post-intervention in both male and female participants, in contrast to the control group. Additionally, female participants in the intervention group demonstrated a significant enhancement in mental health outcomes compared to the control group. The results of this study align with prior research regarding the efficacy of technology-driven interventions in facilitating physical activity and enhancing mental well-being. The investigation additionally recognized a potential disparity between genders in the impacts of the intervention on mental health results, underscoring the necessity for additional research to investigate this disparity and ascertain potential underlying mechanisms. One plausible explanation for the observed gender disparity could be attributed to variations in the nature of physical activities undertaken by male and female individuals, which might yield distinct impacts on mental health outcomes. Additional investigation is warranted to delve into this hypothesis and ascertain potential approaches to enhance the efficacy of technology-driven interventions for enhancing mental health outcomes in individuals of both genders.

The practical implications of these findings hold significance for both researchers and healthcare practitioners. The study provides additional empirical evidence supporting the effectiveness of technology-based interventions in promoting physical activity and improving mental health outcomes. It also highlights the potential gender-related disparities in the impact of these interventions on mental health, underscoring the need for further exploration in this area.

In practical terms, healthcare professionals should consider incorporating technology-driven interventions into their treatment strategies to enhance physical activity and bolster mental health outcomes among their patients. In essence, these results offer valuable insights into the potential of technology-driven approaches to facilitate physical activity and elevate mental well-being. Additionally, they emphasize the importance of future investigations to scrutinize gender-based variations in intervention effectiveness. These practical implications empower healthcare professionals to refine their treatment strategies, ultimately aiming for improved physical activity levels and better mental health outcomes for their patients.

### Limitations and suggestions for further studies

The study has offered valuable insights into the relationship between gender, technology-based physical activity interventions, and their impact on physical activity levels and mental health in workplace settings. However, it is important to acknowledge certain limitations and identify areas for future research. One notable limitation is the potential for limited generalizability. The study’s findings may not fully represent the diversity of workplaces and employee populations, which can vary significantly. Replicating these findings across a range of workplace settings would enhance their applicability. Additionally, the reliance on self-reported data for assessing physical activity levels and mental health outcomes can introduce biases, such as recall and social desirability bias. Future research could benefit from incorporating objective measures like wearable fitness trackers or clinical assessments to provide more accurate and reliable data.

The cross-sectional design of the study, while informative, restricts its ability to establish causality. Longitudinal studies or intervention-based research would offer a more comprehensive understanding of how technology-based interventions influence physical activity and mental health outcomes over time. While the study identified important barriers to technology-based interventions, it may not have captured the full spectrum of obstacles faced by employees in diverse workplace environments. Future investigations should aim to explore a broader range of barriers, including those related to cultural and organizational factors. Additionally, it is worth noting that the study did not extensively examine potential interaction effects between gender and other factors (e.g., age, job role) on motivation and barriers. Exploring these interactions could provide deeper insights into how various factors intersect to influence the effectiveness of technology-based interventions.

Finally, investigating the long-term mental health effects of these interventions and complementing quantitative data with qualitative insights, such as interviews or focus groups, could offer a more comprehensive understanding of employees’ motivations and experiences. In conclusion, while the study makes valuable contributions, these considerations point to areas for future research. Addressing these limitations and exploring these areas further can refine our understanding of how gender, technology-based physical activity interventions, and workplace dynamics interact to impact employee well-being.

### Electronic supplementary material

Below is the link to the electronic supplementary material.

Supplementary Material 1

## Data Availability

The data will be made available upon the request from the corresponding author (Ruilin Xu, email: xrl@zzu.edu.cn).
