# Supplementary material for: The impact of technology on promoting physical activities and mental health: a gender-based study
Source: BMC Psychol. 2023 Sep 29;11:298. doi: 10.1186/s40359-023-01348-3 (PMC10542252; doi:10.1186/s40359-023-01348-3)
Supplement: Supplementary file 1 — Supplementary Material 1 [file 40359_2023_1348_MOESM1_ESM.docx]

**Appendix A**

**Interview questions**

What motivated you to use technology to promote physical activities and mental health?

How did you decide on the specific technology used in your study?

Do you believe that technology can have a significant impact on promoting physical activities and mental health? Why or why not?

How did you recruit participants for your study?

Could you describe the gender-based aspect of your study and why it was important to consider?

Did you encounter any challenges during the implementation of the technology or the conduct of the study? If so, what were they and how did you address them?

**Appendix B**

**Dear participants**

This study aims at investigating the motivations and barriers of using motivations and barriers employees face when using technology-based physical activity interventions in the workplace. The first 8 items are motivations and the last 5 items are barriers. Please use 1 to 5 to state your opinion with each motivation and barrier (1= strongly disagree, 2=disagree, 3= somehow agree, 4= agree, 5= strongly agree)

|  | 1 | 2 | 3 | 4 | 5 |
| --- | --- | --- | --- | --- | --- |
| 1. Technology is used for health and fitness goals |  |  |  |  |  |
| 1. Convenience and accessibility are motivations for using technology in promoting physical activities and mental health |  |  |  |  |  |
| 1. Social support from organization is a motivation for using technology in promoting physical activities and mental health |  |  |  |  |  |
| 1. Incentives and rewards organization is a motivation for using technology in promoting physical activities and mental health |  |  |  |  |  |
| 1. Technology can be used for tracking progress |  |  |  |  |  |
| 1. Each person use organization is a motivation for using technology in promoting physical activities and mental health at their pace |  |  |  |  |  |
| 1. Competition with colleague is an incentive for organization is a motivation for using technology in promoting physical activities and mental health |  |  |  |  |  |
| 1. Through using technological devices stress can be relieved easily. |  |  |  |  |  |
| 1. Lack of time is a barrier to use technology in promoting physical activities and mental health |  |  |  |  |  |
| 1. Lack of motivation is a barrier to use technology in promoting physical activities and mental health |  |  |  |  |  |
| 1. Lack of support is a barrier to use technology in promoting physical activities and mental health |  |  |  |  |  |
| 1. Workplace culture is a barrier to use technology in promoting physical activities and mental health |  |  |  |  |  |
| 1. Technical issues are a barrier to use technology in promoting physical activities and mental health |  |  |  |  |  |
